# Molecular evolutionary analysis of a gender-limited *MID* ortholog from the homothallic species *Volvox africanus* with male and monoecious spheroids

**DOI:** 10.1371/journal.pone.0180313

**Published:** 2017-06-30

**Authors:** Kayoko Yamamoto, Hiroko Kawai-Toyooka, Takashi Hamaji, Yuki Tsuchikane, Toshiyuki Mori, Fumio Takahashi, Hiroyuki Sekimoto, Patrick J. Ferris, Hisayoshi Nozaki

**Affiliations:** 1 Department of Biological Sciences, Graduate School of Science, the University of Tokyo, Bunkyo-ku, Tokyo, Japan; 2 Department of Chemical and Biological Sciences, Faculty of Science, Japan Women's University, Bunkyo-ku, Tokyo, Japan; 3 Department of Tropical Medicine and Parasitology, School of Medicine, Juntendo University School of Medicine, Bunkyo-ku, Tokyo, Japan; 4 College of Life Sciences, Ritsumeikan University, Kusatsu-shi, Shiga, Japan; 5 Department of Ecology and Evolutionary Biology, University of Arizona, Tucson, Arizona, United States of America; Donald Danforth Plant Science Center, UNITED STATES

## Abstract

*Volvox* is a very interesting oogamous organism that exhibits various types of sexuality and/or sexual spheroids depending upon species or strains. However, molecular bases of such sexual reproduction characteristics have not been studied in this genus. In the model species *V*. *carteri*, an ortholog of the *minus* mating type-determining or *minus* dominance gene (*MID*) of isogamous *Chlamydomonas reinhardtii* is male-specific and determines the sperm formation. Male and female genders are genetically determined (heterothallism) in *V*. *carteri*, whereas in several other species of *Volvox* both male and female gametes (sperm and eggs) are formed within the same clonal culture (homothallism). To resolve the molecular basis of the evolution of *Volvox* species with monoecious spheroids, we here describe a *MID* ortholog in the homothallic species *V*. *africanus* that produces both monoecious and male spheroids within a single clonal culture. Comparison of synonymous and nonsynonymous nucleotide substitutions in *MID* genes between *V*. *africanus* and heterothallic volvocacean species suggests that the *MID* gene of *V*. *africanus* evolved under the same degree of functional constraint as those of the heterothallic species. Based on semi quantitative reverse transcription polymerase chain reaction analyses using the asexual, male and monoecious spheroids isolated from a sexually induced *V*. *africanus* culture, the *MID* mRNA level was significantly upregulated in the male spheroids, but suppressed in the monoecious spheroids. These results suggest that the monoecious spheroid-specific down regulation of gene expression of the *MID* homolog correlates with the formation of both eggs and sperm in the same spheroid in *V*. *africanus*.

## Introduction

**Fig 1:**
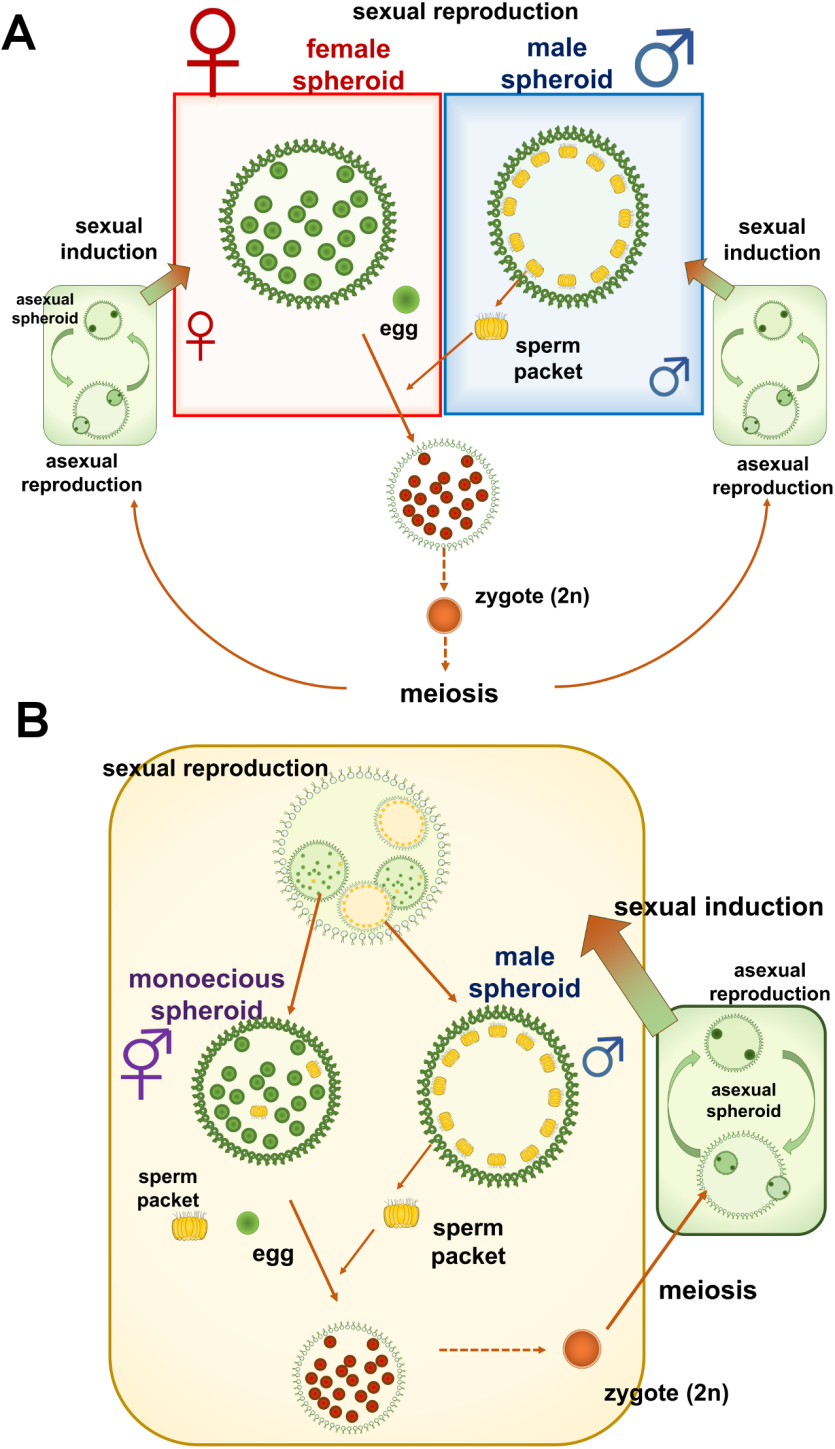
Life cycle diagrams for two related species of *Volvox*. Based on Nozaki et al.[3]. (A) *V*. *reticuliferus* (heterothallic, dioecious type). Germination of a diploid zygote yields a single meiotic product: either a female or a male depending on which MT locus is inherited. The algae reproduce asexually and can undergo sexual induction to produce sperm packets (in male lineages) or eggs (in female lineages). (B) *V*. *africanus* (homothallic, monoecious with males type). Germination of a diploid zygote yields a single meiotic product that reproduces asexually. Upon sexual induction, a clonal population can produce both spheroids containing sperm packets only (male spheroids) and spheroids containing both eggs and sperm packets (monoecious spheroids).

*Volvox* is a genus of spheroidal, multicellular green algae with a surface layer of hundreds to thousands of biflagellated somatic cells, and a much smaller number of non-flagellated germ cells (gonidia) that develop into asexual progeny. Life cycles of all *Volvox* species are facultatively sexual with haploid asexual phase; typically many rounds of asexual reproduction occur between rounds of sexual reproduction in which thick-walled diploid zygotes are formed and meiosis occurs during zygote germination to produce haploid progeny (Fig 1). During sexual reproduction, spheroids that contain eggs or sperm packets (bundles of male gametes or sperm) or both are produced. This genus exhibits various types of sexuality and/or sexual spheroids that have been used to define separate taxa within *Volvox* [1–3]. For example, whether the sexual spheroids are dioecious or monoecious is an important criterion for distinguishing species of *Volvox*; several monoecious species are recognized in *Volvox* [1–3].

Starr [4] recognized four types of sexuality in several strains identified as *Volvox africanus* originating from locations around the world (S1 Fig); 1) heterothallic, dioecious type: male spheroids (containing sperm packets) or female spheroids (containing eggs) are formed in the male or female strain, respectively; 2) homothallic, dioecious type: separate male and female spheroids are formed in the same strain; 3) homothallic, monoecious type: monoecious spheroids (containing both eggs and sperm packets) are formed; and 4) homothallic, monoecious with males type: monoecious spheroids and male spheroids are both formed in the same strain. Coleman [5] resolved a small clade composed of these four sexual types of *V*. *africanus* based on the internal transcribed spacer-2 (ITS-2) of nuclear ribosomal DNA (rDNA) sequences. Thus, these related strains may be very useful for studying the diversity and evolution of monoecy and/or homothallism in *Volvox*. However, further studies of sexuality in these strains have been lacking except for the heterothallic, dioecious type [6], since strains exhibiting the three types of homothallic sexuality are not available [3]. Recently, new Japanese strains of two *V*. *africanus*-like species were isolated from water samples collected in Lake Biwa, Japan [3]. One that corresponds to sexual type 1 (heterothallic, dioecious type) by Starr [4] was renamed as a new species, *V*. *reticuliferus* (Fig 1A, S2D and S2E Fig). The other was re-identified as *V*. *africanus*, and produces both monoecious and male spheroids in a single strain (sexual type 4 of Starr [4], Fig 1B and S2 Fig).

In the heterothallic isogamous species *Chlamydomonas reinhardtii*, two sexes or mating types are determined by the presence or absence of the mating type-specific minus dominance gene (*MID*) [7]. In anisogamous volvocine *Pleodorina starrii* and oogamous *Volvox carteri*, a *MID* ortholog is present only in male strains [8,9]. Although *MID* is the master gene determining mating type minus of *C*. *reinhardtii* [7], the *MID* ortholog in *V*. *carteri* (*VcMID*) was recently reported as regulating formation of sperm packets, but not formation of male-specific sexual spheroids [8–10]. The *MID* ortholog is present in only one of the two heterothallic mating types in the isogamous volvocine *Gonium*, but it is present in a homothallic strain of *Gonium multicoccum* [11].

In the male strain of heterothallic *V*. *carteri*, experimental knock-down of *VcMID* results in sexual spheroids with eggs and sperm packets (similar to monoecious spheroids in wild monoecious species) or female-like sexual spheroids (with eggs and no sperm packets), depending upon the degree of suppression of *VcMID* expression [10]. This implies *MID* gene expression may be important for formation of monoecious spheroids in homothallic wild species of *Volvox*. However, *MID* orthologs in wild homothallic *Volvox* species with monoecious spheroids, like sexual type 3 or 4 of Starr [4], have not been previously reported.

To understand the evolution and development of monoecious spheroids in wild *Volvox* species, comparative analysis of their *MID* genes with those of closely related heterothallic dioecious species should be fruitful. In the present study, we examined *MID* homologs from two closely related species of *Volvox* sect. *Merrillosphaera*: *V*. *africanus* and *V*. *reticuliferus*, identified by Nozaki et al.[3]. Since these two species are heterothallic, dioecious type and homothallic, monoecious with males type (sexual types 1 and 4 by Starr [4], respectively), comparative analyses of *MID* orthologs from these two species will lead to a greater understanding of the evolution of monoecy or homothallism in *Volvox*. In addition, in order to elucidate general *MID* characteristics of monoecious spheroids, the *MID* homolog from *V*. *ferrisii* which produces only monoecious sexual spheroids [2] was also studied. *V*. *ferrisii* is belongs to *Volvox* sect. *Volvox* that is phylogenetically separated from *Volvox* sect. *Merrillosphaera* [3].

## Materials and methods

### Strains and culture conditions

#### *Volvox africanus* and *V*. *reticuliferus*

*Volvox africanus* strain 2013-0703-VO4 (= NIES-3780) and six strains of *V*. *reticuliferus* (Table 1) were used in the present study. The cultures were maintained in screw-cap tubes (18 × 150 mm) containing 10 ml AF-6/3 medium [3] at 20°C on a 14-h light: 10-h dark schedule or at 25°C on a 12-h light:12-h dark schedule under cool-white fluorescent lamps at an intensity of 55–80 μmol∙m^–2^∙s^–1^.

**Table 1:** Strains of the three *Volvox* species used in the present study.

Species	Strain designation	Origin	Sex	Reference
*V*. *africanus*	2013-0703-VO4	Lake Biwa, Japan	Monoecious with males	Nozaki et al. [3]
	(= NIES-3780)			
*V*. *reticuliferus*	2013-0703-VO2	Lake Biwa, Japan	Female	Nozaki et al. [3]
	(= NIES-3782)			
	2013-0703-VO3	Lake Biwa, Japan	Male	Nozaki et al. [3]
	(= NIES-3783)			
	VO123-F1-6	F_1_ progeny strain of 2013-0703-VO1 x VO2 x VO3	Female	Nozaki et al. [3]
	(= NIES-3785)			
	VO123-F1-7	F_1_ progeny strain of 2013-0703-VO1 x VO2 x VO3	Male	Nozaki et al.[3]
	(= NIES-3786)			
	VO123-F1-9	F_1_ progeny strain of 2013-0703-VO1 x VO2 x VO3 (sibling strain of NIES-3785 and NIES-3786)	Female	Nozaki et al. [3]
	(= NIES-4110)			
	VO123-F1-10	F_1_ progeny strain of 2013-0703-VO1 x VO2 x VO3 (sibling strain of NIES-3785 and NIES-3786)	Male	Nozaki et al. [3]
	(= NIES-4111)			
*V*. *ferrisii*	2011-929-Vx2-F2-9	F_2_ progeny of NIES-2737	Monoecious	Isaka et al. [2]
	(= NIES-3986)			

To induce sexual reproduction, about 0.5 ml of growing cultures were transferred into 10 ml of USVT medium diluted one to two with distilled water [3] and grown at 25°C on a 12-h light:12-h dark schedule under cool-white fluorescent lamps at an intensity of 160–180 μmol∙m^–2^∙s^–1^. Sexual spheroids developed after 4~5 days (*V*. *africanus*) or 7~10 days (*V*. *reticuliferus*).

#### Volvox ferrisii

*Volvox ferrisii* strain 2011-929-Vx2-F2-9 (= NIES-3986) was cultured in screw-cap tubes containing 10 ml AF-6 medium [12,13] at 20°C on a 14-h light: 10-h dark schedule under cool-white fluorescent lamps at an intensity of 55–80 μmol∙m^–2^∙s^–1^.

To induce sexual reproduction, about 0.5 ml of growing cultures were transferred into 10 ml of VTAC medium [13,14] and grown at 25°C on a 12-h light:12-h dark schedule under cool-white fluorescent lamps at an intensity of 160–180 μmol∙m^–2^∙s^–1^. After 7~10 days sexual spheroids developed abundantly.

### Identification of *MID* orthologs

#### V. africanus

A full-length sequence of the *V*. *africanus MID* (*VaMID*) mRNA was determined from total RNA using RT-PCR with degenerate primers (S1 Table) as described previously [9,15]. Total RNA was isolated from cultures in which sexual reproduction had been induced as described above, using the RNeasy Mini Kit (Qiagen, Hilden, Germany) after the cells had been homogenized with ceramic beads and a wash brush [9,15]. Production of cDNA was carried out with Superscript III reverse transcriptase (Thermo Fisher Scientific, MA, USA) using 3'-RACE CDS Primer A from the SMARTer™ RACE cDNA Amplification Kit (Clontech Laboratories, Inc., CA, USA). Nested PCR using this cDNA as template with degenerate *MID*-gene primers (S1 Table) yielded a partial fragment of *VaMID*. The primers used in in the first PCR were dMT-dF3 [16] (S1 Table) and Nested Universal Primer A (Clontech Laboratories); the primers used in the second PCR were dMT-dF3 and SMID-R6. The PCR reactions were carried out using rTaq polymerase (TAKARA, Shiga, Japan) and the cycling conditions described previously [17]. The resulting fragments were TA subcloned using the TOPO TA Cloning Kit (Thermo Fisher Scientific), and the plasmid insert sequenced on an ABI PRISM 3100 Genetic Analyzer (Thermo Fisher Scientific) using the BigDye Terminator Cycle Sequencing Ready Reaction Kit v. 3.1 (Thermo Fisher Scientific) as described previously [9].

To determine the 3’-terminus sequence of *VaMID*, 3’-RACE was performed with Nested Universal Primer A and gene specific primers VaMID_F1, VaMID_F2 and VaMID_F3 (S2 Table). The resulting fragments were TA subcloned using the TOPO TA Cloning kit, and sequenced as described above. The 5’-terminus sequence was determined using the GeneRacer kit (Thermo Fisher Scientific) according to the manufacturer’s protocol; the antisense gene specific primer was VaMID_5’R1 (S2 Table). Nested PCR was performed with GeneRacer 5’ Primer and gene specific primers VaMID5'R1, VaMID5'R2 and VaMID5'R3 (S2 Table). The resulting fragments were TA subcloned and sequenced as described above.

To determine the intron-exon structure of *VaMID*, genomic PCR using total DNA extracted as described previously [18] was performed, followed by DNA sequencing of the product. The PCR reaction used KOD FX Neo DNA polymerase (TOYOBO, Osaka, Japan) and *VaMID*-specific primers (VaMID_AR and VaMID_ValR2; S2 Table) with cycling conditions 2 min at 94°C, followed by 35 cycles of 10 sec at 98°C and 30 sec at 68°C.

#### V. ferrisii

A partial sequence of *V*. *ferrisii MID* (*VfMID*) mRNA was obtained by PCR amplification and sequencing as described for *VaMID* except for the primers used for the nested PCR and determination of 3’ and 5’ termini (S2 Table). Degenerate primers SMID-F1 and SMID-R5 were used for the first PCR, and SMID-F1 and SMID-R4 for the second PCR (S1 Table). To determine the 3’-terminus sequence of *VfMID*, 3’-RACE was performed with Nested Universal Primer A and *VfMID*-specific primers VfMID_F1, VfMID_F2, VfMID_F3, VfMID_F4, VfMID_F5 and VfMID_F6 (S2 Table). Specific primers VfMID_R1 and VfMID5'R (S2 Table) were used for amplifying the 5’-terminus sequence.

The intron-exon structure of *VfMID* was determined using genomic PCR as described above for *VaMID* but using *VfMID*-specific primers (VfMID_Af and VfMID_AR; S2 Table).

#### V. reticuliferus

Polyadenylated mRNA was isolated from sexually induced cultures using Dynabeads Oligo (dT)_25_ (Thermo Fisher Scientific) and reverse transcribed with Superscript III reverse transcriptase (Thermo Fisher Scientific).

We performed 5’- and 3’-RACE with the GeneRacer kit and *V*. *reticuliferus MID* (*VrMID*) specific primers based on the partial *MID* sequences of *V*. *reticuliferus* strain UTEX 1890 [3,6]. 5’ nested PCR was performed with Gene Racer 5’ Primer and gene specific primers F1-7MID_R1 and F1-7MIDR2 (S2 Table). 3’ PCR were performed using the Gene Racer 3’ Primer and a gene specific primer, F1-7MID_3’F1 (S2 Table).

The intron-exon structure of *VrMID* was determined using genomic PCR as described above for *VaMID* but using *VrMID*-specific primers (F1-7MID_AF and F1-7MID_AR; S2 Table).

#### *Yamagishiella unicocca* and *Eudorina* sp.

Full-length *MID* genes of *Y*. *unicocca* strain NIES-1859 and *Eudorina* sp. strain NIES-2735 (S3 Table) were determined as described previously [9,16].

#### Availability of sequence data

The new sequence data of *MID* orthologs have been deposited to DDBJ/EMBL/GenBank (accession numbers: LC274875-LC274882; S3 Table).

### Phylogenetic analysis of *MID* orthologs

Phylogenetic analyses were performed using MUSCLE [19]–aligned full-length protein sequences of sixteen Volvocales MID orthologs (S3 Table). The maximum likelihood (ML) method (based on LG model [20] selected by MEGA 6.0 [21]) and the neighbor joining (NJ) method (using JTT model [22]) by MEGA 6.0) were carried out with bootstrap values from 1000 replications.

A molecular evolutionary analysis of nonsynonymous and synonymous substitutions was performed between the *MID* ortholog of *G*. *pectorale* and those of seven other Volvocales by MEGA 6.0, using a modified Nei-Gojobori model [23,24] (assumed transition/transversion bias = 1.55).

### Detection of *VrMID* based on genomic PCR of *V*. *reticuliferus*

Genomic PCR was performed in six strains of *V*. *reticuliferus* (Table 1) using total DNA extracted as described previously [18], KOD FX Neo DNA polymerase and a pair of *VrMID* specific primers (F1-7MID_AF and F1-7MID_AR; S2 Table). The ITS2 sequence was amplified as a control, using an ITS2-specific primer pair designed based on the ITS2 sequence of 2013-0703-VO2 (S2 Table). PCR cycles were 2 min at 94°C, followed by 30 (ITS2) and 35 (*VrMID*) cycles of 10 sec at 98°C and 30 sec at 68°C.

### Southern blot analysis

**Fig 2:**
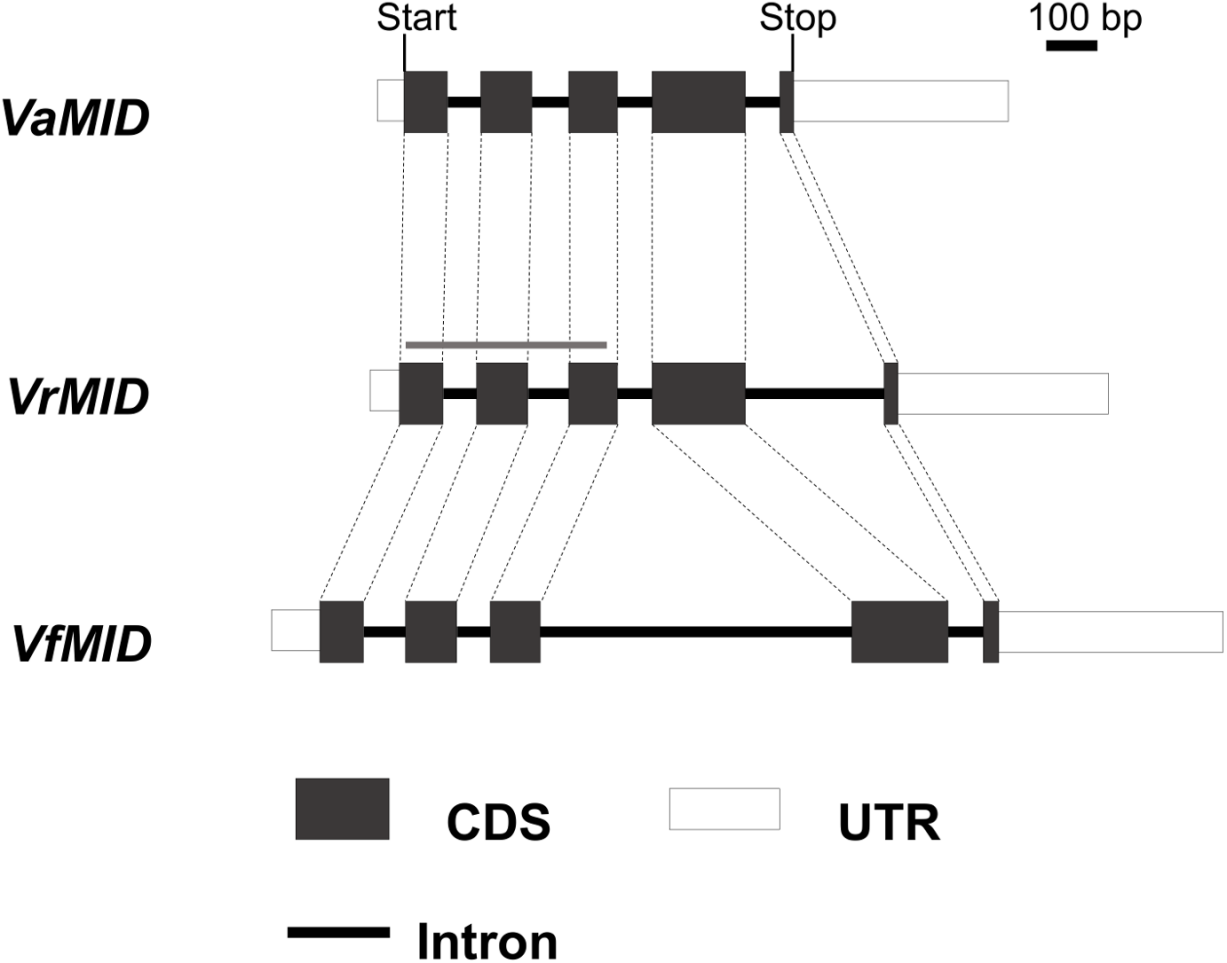
Exon-intron structures of three *MID* orthologs: *VaMID* (*Volvox africanus*), *VrMID* (*V*. *reticuliferus*), and *VfMID* (*V*. *ferrisii*). Gray bar represents the *VrMID* probe for Southern blotting (S5 Fig).

Genomic DNA of *V*. *africanus* strain 2013-0703-VO4, *V*. *reticuliferus* strains VO123-F1-6 (female) and VO123-F1-7 (male) (Table 1) was prepared by the “miniprep” method [25]. Restriction enzyme digests of genomic DNA (2 μg) were separated by 1.0% agarose gel electrophoresis and transferred onto a Hybond-N+ nylon membrane (GE Healthcare, UK). A hybridization probe containing part of the *VrMID* gene (Fig 2) labeled with digoxigenin-11-dUTP was prepared by PCR using a plasmid clone of the *VrMID* gene as template and the primer pair F1-7_southMID_F and F1-7_MID_R1 (S2 Table) using PCR DIG Probe Synthesis Kit (Roche Diagnostics, Germany), and hybridized at 42°C. A hybridization probe containing part of the *V*. *reticuliferus* elongation factor 1-like gene (*EF1-like*) labeled with digoxigenin-11-dUTP was prepared by PCR using a plasmid clone of the *EF1-like* gene as template and the primer pair (CV_EF1A1-R2 and GpEF1A-INT3-R [16]; S2 Table) using KOD FX Neo DNA Polymerase, and hybridized at 42°C. The signals were detected using DIG-High Prime DNA Labeling and Detection Starter Kit II (Roche Diagnostics) and Chemidoc XRS (Bio-Rad, Hercules, CA, USA). The resulting image was processed with a median filter (diameter: 1 pixel) in ImageJ (National Institutes of Health, Bethesda, MD, USA) to remove random noise produced by long exposure (2 hr).

### Estimation of genome sizes of *Volvox africanus* and *V*. *reticuliferus*

To estimate relative genome size of *V*. *africanus* and *V*. *reticuliferus*, 4’,6-diamidine-2-phenylindole (DAPI)-staining was performed using somatic cells of *V*. *africanus*, male and female strains of *V*. *reticuliferus* (2013-0703-VO2 and 2013-0703-VO3, respectively), and *V*. *carteri* strain EVE (control). One ml of each vegetative sample was fixed with 0.25% glutaraldehyde, followed by postfixation in 100% methanol for reducing autofluorescence, and washed with phosphate-buffered saline. Fixed samples were stained with 0.1μg/μl DAPI overnight. DAPI-stained somatic cells of *V*. *africanus* and *V*. *reticuliferus* male and female were mixed separately with DAPI-stained *V*. *carteri* strain EVE and mounted in the same slide. The images were obtained using a BX-60 Microscope and DP Controller 1. 2. 1108 (Olympus, Tokyo, Japan). The image analyses were performed using ImageJ, measuring the mean gray value of 10 nuclei for each exposure time (0.50, 0.67, 1.0, 1.5, 2.0 and 2.5 s).

### Semi-quantitative reverse transcription (RT)-PCR analyses

*EF1-like* genes were used as internal controls. To obtain sequences of *EF1-like* genes from the three *Volvox* species, PCR amplifications were performed with full-length cDNA of each *Volvox* and the primer pair, CV_EF1A1-R2 and GpEF1A-INT3-R [16]. From direct sequencing of PCR-products, we designed *EF1-like*-specific primer pairs for each of the three *Volvox* species (S2 Table) for semi-quantitative RT-PCR analyses.

For *V*. *africanus*, 30 asexual, male or monoecious spheroids were collected by a micropipette from cultures that were sexually induced or not. Polyadenylated mRNAs were isolated separately from these three sets of spheroids and reverse transcribed as described for *V*. *reticuliferus MID* determination. Likewise, cDNAs of the other two *Volvox* species were obtained by reverse transcription using mRNAs isolated from 30 asexual (in cultures that were sexually induced or not), male or female spheroids in *V*. *reticuliferus*, as well as from 30 asexual (in cultures that were sexually induced or not), or monoecious spheroids in *V*. *ferrisii*.

PCR analyses were performed using KOD FX Neo DNA polymerase. PCR cycles and primer pairs are described in S4 Table. The amplified products were electrophoresed on 2% (wt/vol) agarose gels and stained with ethidium bromide. The gel images were captured using a ChemiDoc XRS (Bio-Rad), level adjusted and gradation inverted with Adobe Photoshop CS6 (Adobe Systems Inc., San Jose, CA).

## Results

### Identification and characterization of *MID* orthologs

We identified full-length cDNA sequences and intron-exon structures of *VaMID*, *VrMID* and *VfMID* (Fig 2). The genomic sequences of the three genes determined in this study covered the entire DNA sequences of the genes and demonstrated that all three genes contained introns at the same four positions. The deduced protein sequences of the three genes were composed of 163–167 amino acids that contained the DNA binding RWP-RK domain near the C-terminus. RWP-RK domains of seven volvocine MID proteins were highly conserved even among homothallic and heterothallic volvocine species (S3 Fig).

**Fig 3:**
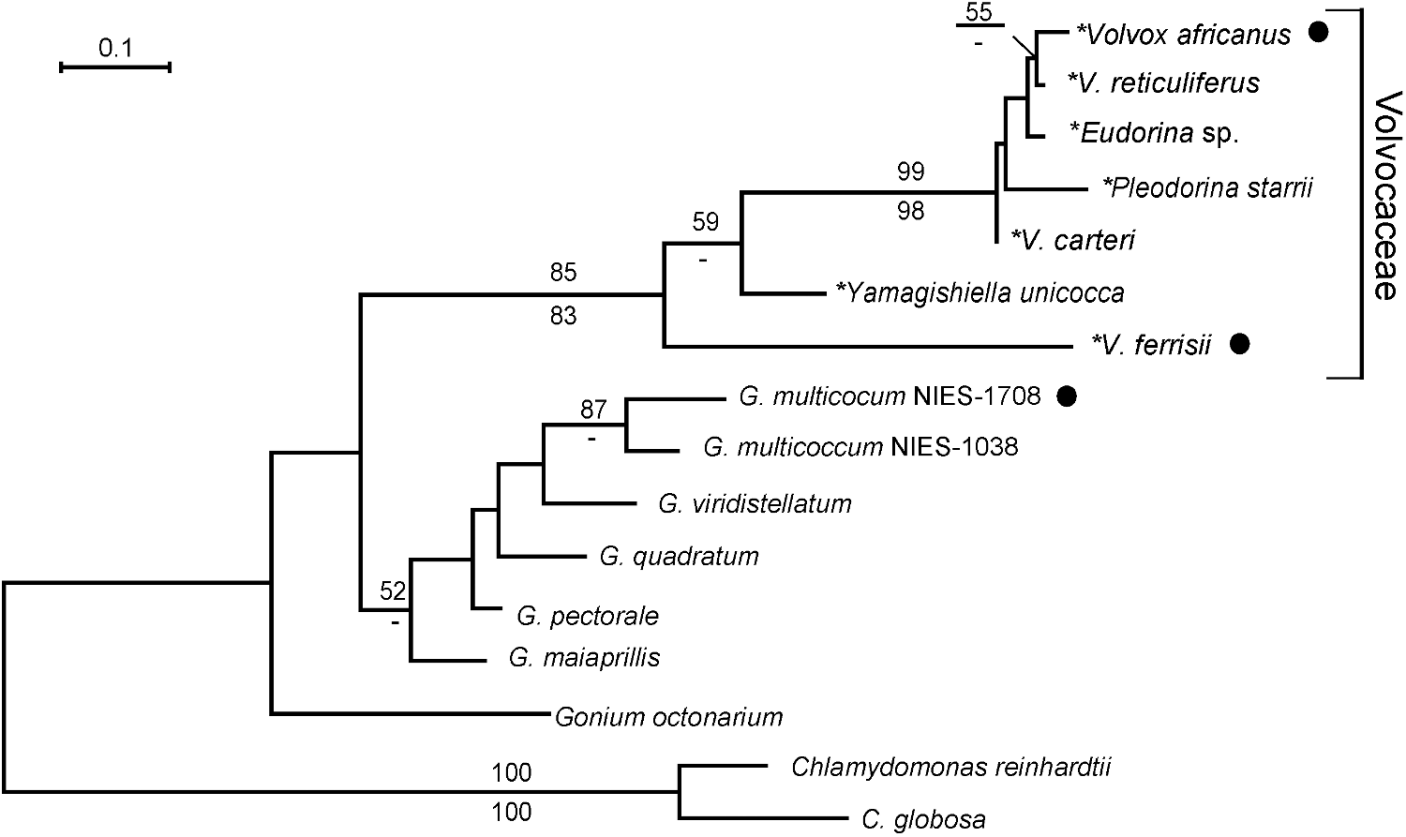
Maximum-likelihood (ML) tree (based on LG model) of 16 full-length MID proteins from colonial volvocine species and two species of *Chlamydomonas*. Branch lengths are proportional to the estimated amino acid substitutions, which are indicated by the scale bar above the tree. Numbers above and below branch points indicate bootstrap values (50% or more) of the ML and neighbor-joining (based on the JTT model), analyses, respectively. The sequences of *MID* orthologs with asterisks (*) were determined in this study; filled circles (●) indicate homothallic strains.

Based on the phylogenetic analysis of 14 colonial volvocine MID proteins, a large clade composed of seven genes of the Volvocaceae was resolved with 83–85% bootstrap values in ML and NJ methods (Fig 3). However, phylogenetic relationships of *Gonium* MID proteins were not well resolved. Within the volvocacean clade, MID proteins from *V*. *carteri*, *V*. *reticuliferus*, *V*. *africanus*, *Pleodorina starrii* and *Eudorina* sp. formed a robust monophyletic group (with 98–99% bootstrap values in both analyses) from which *Yamagishiella unicocca* and *V*. *ferrisii* MID proteins were separated. These results were consistent with the phylogenetic relationships of the colonial volvocine algae based on chloroplast genes [26].

**Fig 4:**
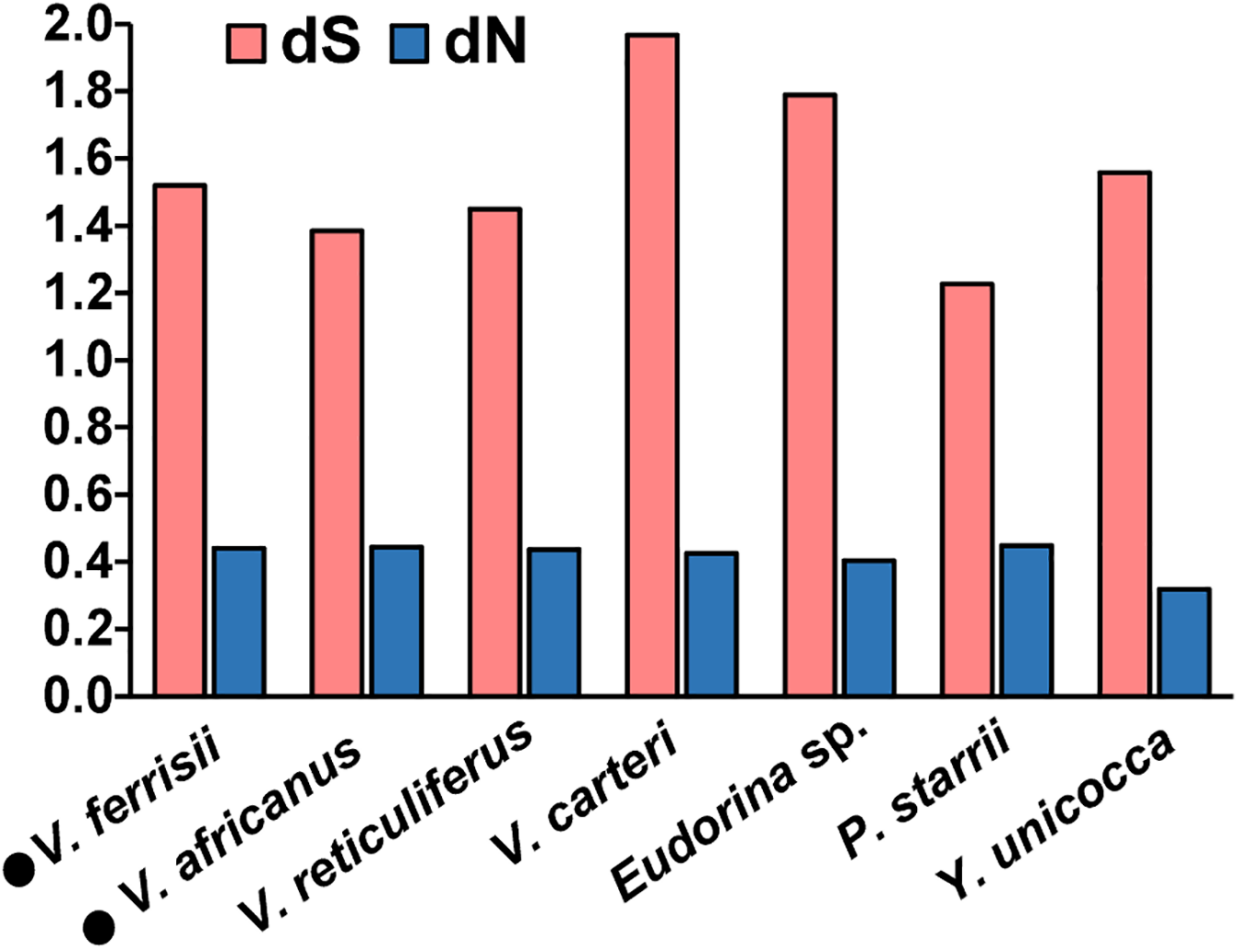
Synonymous (dS) and nonsynonymous (dN) substitutions of *MID* genes in the Volvocaceae of volvocine algae (Fig 2). Analyses were conducted with the outgroup *Gonium pectorale MID* gene (AB353340) using the modified Nei-Gojobori (assumed transition/transversion bias = 1.55) model ([23,24] by MEGA6[21]. All positions containing gaps and missing data were eliminated. There were a total of 162 positions in the final dataset. Filled circles (●) indicate the homothallic strain.

A molecular evolutionary analysis of the volvocacean *MID* genes demonstrated that nonsynonymous and synonymous substitutions of the genes from two homothallic species of *Volvox* (*V*. *africanus* and *V*. *ferrisii*) fell within the range of those of heterothallic species (Fig 4).

### Genomic PCR and Southern blot analysis of *MID* genes of *Volvox africanus* and *V*. *reticuliferus*

Results of genomic PCR using *VrMID*-specific primers (S2 Table) for strains of *V*. *reticuliferus* (Table 1) are shown in S4 Fig. All three male strains of *V*. *reticuliferus* demonstrated the presence of *VrMID* based on a single band whereas all three female *V*. *reticuliferus* strains lacked the gene. Four of the *V*. *reticuliferus* strains are F1 progeny in which the *MID* gene band is found only in phenotypically male strains. This is consistent with the expectation that a *MID* gene containing MT locus is the genetic determiner of sex, although more progeny are needed to be definitive. In the homothallic species *V*. *africanus*, a single band of *VaMID* was detected (not shown).

Southern blot analysis of *V*. *reticuliferus* demonstrated the presence of a single copy of the *VrMID* gene in the male genome and the complete absence of the gene in the female (S5A Fig). The genome of the homothallic species *V*. *africanus* was shown to encode two possible copies of *VaMID* based on the blot analysis (S5A Fig). However, only a single copy of *EF1-like* gene was detected in each strain of *V*. *africanus* and *V*. *reticuliferus* (S5B Fig).

### Estimation of genome sizes of *Volvox africanus* and *V*. *reticuliferus* based on epifluorescence microscopy of DAPI-stained somatic cells.

Since *V*. *africanus* or *V*. *reticuliferus* might have originated from their common ancestor by duplication of the whole genome, relative genome sizes of these two species were measured based on the degree of fluorescence of DAPI-stained nuclei in somatic cells using epifluorescence microscopy. By using the fluorescence value of nuclei of *V*. *carteri* EVE somatic cells as a control, both *V*. *africanus* and *V*. *reticuliferus* genome sizes could be considered to be 0.9–1.1 times the genome size of *V*. *carteri* EVE (S6 Fig).

### Semi-quantitative RT-PCR analyses of expression of *MID* genes

**Fig 5:**
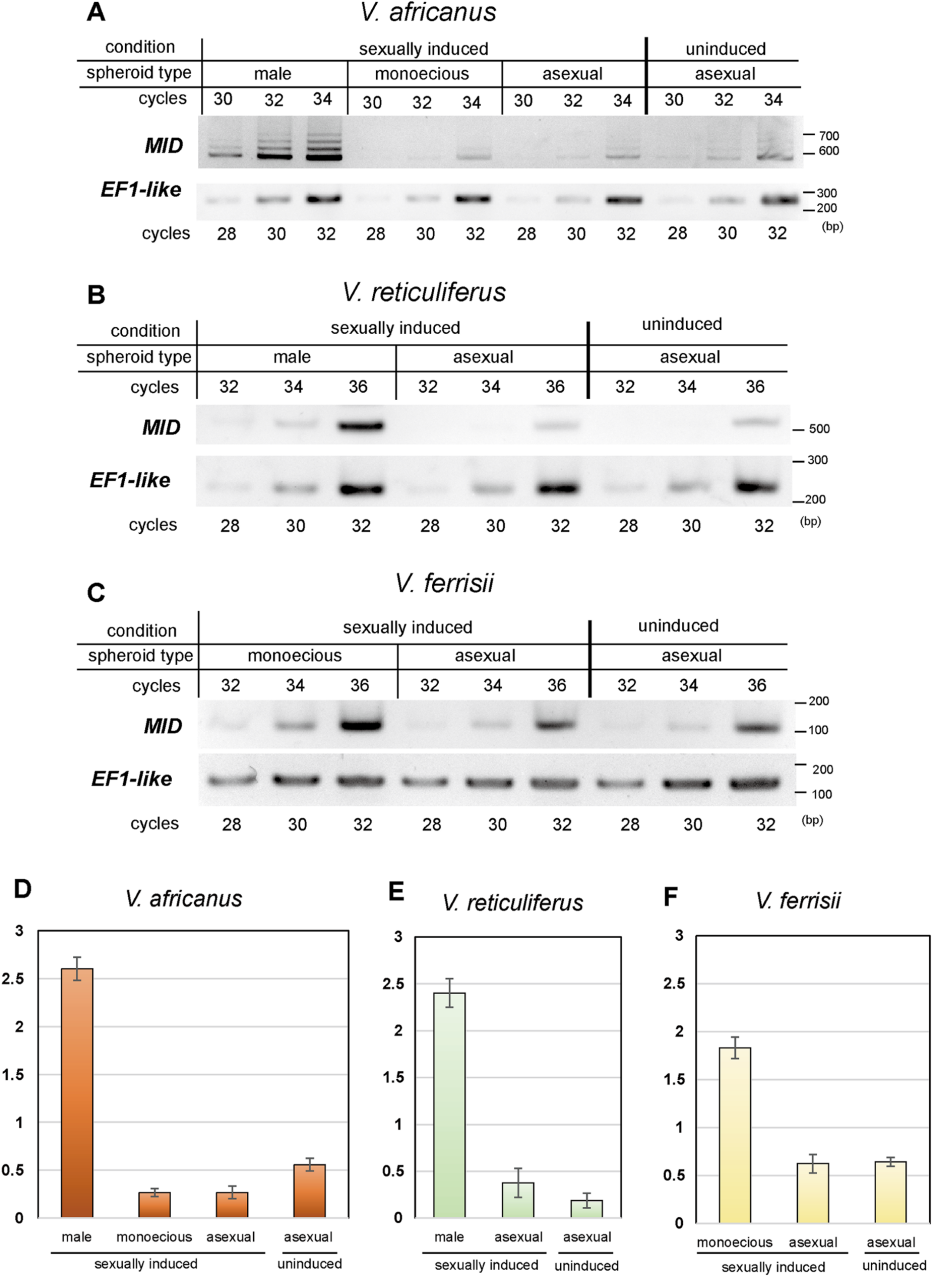
Semi-quantitative RT-PCR of *MID* orthologs in three species of *Volvox* (*V*.). **(**A-C) The products of RT-PCR reactions are resolved by agarose gel electrophoresis. The loading volume for each lane was normalized to the quantity of *EF1-like* (internal control) product. The number of PCR cycles are indicated separately for *MID* and *EF1-like*. (D-F) Gel band quantification analyses by ImageJ. Bars show means and standard deviations of three individual experiments.

Results of semi-quantitative RT-PCR analyses of expression of *MID* genes in the *V*. *africanus* homothallic strain, *V*. *reticuliferus* male strain and *V*. *ferrisii* homothallic strain are shown in Fig 5. In a sexually induced *V*. *africanus* culture, *VaMID* expression was extremely high in male spheroids, whereas the expression was low in monoecious and asexual spheroids (Fig 5A and 5D). The *VaMID* expression of asexual spheroids in a sexually uninduced culture was slightly higher than that in a sexually induced culture (Fig 5A). In heterothallic *V*. *reticuliferus*, the *VrMID* level was highly upregulated in male spheroids when compared to that of asexual spheroids in the same culture (Fig 5B and 5E). Three alternative splicing variants of *VaMID* were also identified. All these variants were identified as due to intron retention [27] (S7 Fig). No alternative splicing variants were identified in *VrMID* and *VfMID*. In contrast to *V*. *africanus*, *VrMID* expression of asexual spheroids in a sexually induced culture was slightly higher than that in a sexually uninduced culture (Fig 5E). No alternative splicing variants were detected in *VrMID* expression.

In contrast to *V*. *africanus*, the *VfMID* level in monoecious spheroids of *V*. *ferrisii* was higher than that of asexual spheroids in sexually induced and sexually uninduced cultures. The *VfMID* transcription level in monoecious spheroids was more than 2.5 times higher than that of asexual spheroids from either culture condition.

## Discussion

### *MID* orthologs in homothallic species of *Volvox* with monoecious spheroids

The present study demonstrated that two homothallic species of *Volvox* with monoecious spheroids, *V*. *africanus* and *V*. *ferrisii*, have *MID* orthologs (Figs 2 and 3). The *MID* orthologs (*VaMID* and *VfMID*) of these two homothallic species are essentially consistent with those of heterothallic colonial or multicellular volvocacean species [8,9] in containing 5 exons, 4 introns, and a DNA binding RWP-RK domain at the C-terminus. Phylogenetic relationships of *MID* orthologs within the Volvocaceae (Fig 3) were consistent with those based on chloroplast genes [2,3]. Moreover, comparison of synonymous and nonsynonymous substitutions of *MID* genes between homothallic and heterothallic volvocacean species suggested that the *MID* genes of the two homothallic species have evolved under the same degree of functional constraint as those of the heterothallic species. Thus, no signs of altered selection on *MID* could be detected in the monoecious species. Nozaki et al. [9] reported that MID protein expression is strong in nuclei of the gametes of the male strain of *P*. *starrii*. Geng et al. [10] demonstrated that the *MID* ortholog (*VcMID*) of the heterothallic species *Volvox carteri* controls sperm packet formation by sexual reproductive cells (androgonidia). The present study showed that expression of *VaMID* in homothallic *V*. *africanus* is very high in male spheroids (Fig 5A and 5D). Therefore, the *MID* orthologs of the two homothallic species of *Volvox* may control sperm packet formation as in the heterothallic species.

### *VaMID* transcription in monoecious and male spheroids

The number of sperm packets in a monoecious spheroid is very small, 1–4 in *V*. *africanus* [3] or 3–5 sperm packets in *V*. *ferrisii*. [2]. In contrast, the male spheroid of *V*. *africanus* contains 100–260 androgonidia that divide to form sperm packets. The semi-quantitative RT-PCR data showed down-regulation of *MID* expression in monoecious spheroids and extremely high upregulation in male spheroids in *V*. *africanus*, suggesting that *VaMID* transcriptional level is correlated with the quantity of sperm packets in monoecious or male spheroids. It indicates that *V*. *africanus* has spheroid type-specific regulation of *VaMID*. In heterothallic *V*. *carteri*, VcMID protein is localized in the sperm nucleus and controls formation of sperm packets [10]. As discussed above, *VaMID* may also control formation of sperm packets. Thus, *V*. *africanus* may determine the fate of reproductive cells in monoecious spheroids by differentially controlling *VaMID* expression between eggs and androgonidia. Further analyses of the localization of VaMID in the monoecious spheroid at the cellular level is required to confirm this hypothesis.

### *VfMID* transcription in monoecious spheroids

In *V*. *africanus*, expression of *VaMID* in monoecious spheroids is lower than that of asexual spheroids (Fig 5A and 5D). By contrast, *VfMID* expression in monoecious spheroids is higher than that of asexual spheroids (Fig 5C and 5F). This difference of *MID* expression in monoecious spheroids may be related to the phylogenetic positions of *V*. *africanus* and *V*. *ferrisii*. *V*. *ferrisii* belongs to *Volvox* sect. *Volvox* that is clearly separated from the large monophyletic group (Eudorina group) composed of the other three *Volvox* sections (including *V*. *africanus*, *V*. *reticuliferus* and *V*. *carteri*), *Eudorina* and *Pleodorina* [2,3,27]. The phylogenetic positions suggest that monoecious spheroids might have been acquired independently in the evolutions of *Volvox* sect. *Volvox* and the *Eudorina* group. Further studies of *VfMID* expression at the cellular level are needed to understand the role of *VfMID* in the monoecious species *V*. *ferrisii*.

## Conclusions

Sexual differentiation in heterothallic species of the unicellular and colonial/multicellular volvocine algae is controlled by a sex-determining or mating type locus (*MT*) containing *MID* in the *minus* or male strain [8, 28, 29]. Comparative analyses of *MT* loci in volvocine algae are important to elucidate the molecular and genomic basis of evolution of sexual differentiation [8, 29]. However, there is, as yet, no genome information from homothallic species in the chromosomal region homologous to the *MT* locus of their heterothallic relatives, except for the *MID* genes that we described here in two homothallic species, *V*. *africanus* and *V*. *ferrisii*.

A homothallic euascomycete fungus, *Neosartorya fischeri*, has duplicated *MT* loci, (*MAT1* and *MAT2*) possibly originating from the *MT* loci of the complementary sexes of the heterothallic ancestral species [30]. In *C*. *reinhardtii*, diploid heterozygotes (*mt*^+^/*mt*^-^) sometimes skip meiosis and produce diploid vegetative cells under experimental conditions [31]. The present study showed that the genome size in homothallic *V*. *africanus* is almost the same as that of its heterothallic relative *V*. *reticuliferus* (S6 Fig). Thus, the origin of the homothallic *V*. *africanus* cannot be explained simply by whole genome duplication via lack of meiosis of a diploid zygote of a possible heterothallic ancestor, although partial duplication of only the male and female *MT* loci cannot be ruled out based on the present genome measurement (S6 Fig).

Homothallic *V*. *africanus* has an ortholog of the male-limited gene *MID* found in heterothallic species. In addition, androgonidia (male reproductive cells) in the heterothallic, male strain of *V*. *carteri* may function as eggs by experimental suppression of *VcMI*D expression [10]. The present study demonstrated monoecious spheroid-specific down regulation of gene expression of the *MID* ortholog in *V*. *africanus* (Fig 5). Therefore, the homothallic species *V*. *africanus* might have evolved directly from a male strain of the heterothallic ancestor by modification of the regulation system of *MID* expressions in sexual spheroids. In any case, comparative analyses of the whole genomes of *V*. *africanus* and *V*. *reticuliferus* would be indispensable for further understanding the molecular and genome bases of evolution of both species.

The present study suggested that the male-specific transcription factor MID is functional in two homothallic *Volvox* species that produce both eggs and sperm packets in a single sexual spheroid. As discussed above, *MID* in homothallic species of *Volvox* may also be a key gene that controls formation of sperm packets. Thus, other unknown factors controlling *MID* may be crucial for differentiating monoecious or male spheroids in *Volvox*. Further studies of molecular mechanisms controlling *MID* in various sexual types of *Volvox* will improve our understanding of the evolution of monoecious spheroids in *Volvox*.

## Supporting information

S1 Fig The four sexual types of *Volvox africanus*-like algae recognized by Starr (1971, Sexual reproduction in *Volvox africanus*. Contributions in Phycology. Allen Press, pp. 59–66).
(DOCX)

S2 Fig Light microscopic images of *Volvox africanus* (homothallic, monoecious with males type) and *V*. *reticuliferus* (heterothallic, dioecious type). Scale bars = 50 μm. sp: sperm packet, e: egg. A-C. *V*. *africanus* strain 2013-0703-VO4. A. Asexual spheroid. B. Monoecious spheroid. C. Male spheroid. D, E. *V*. *reticuliferus*. D. Male spheroid in male strain VO123-F1-7. E. Female spheroid in female strain VO123-F1-6.
(DOCX)

S3 Fig Alignment of seven MID homologs from the Volvocaceae (*Volvox africanus*, *V*. *reticuliferus*, *V*. *ferrisii*, *V*. *carteri*, *Eudorina* sp., *Pleodorina starrii*, and *Yamagishiella unicocca*). Black or gray back colors indicate over 70% of identity or similarity, respectively.
(DOCX)

S4 Fig Results of genomic PCR for parental strains and four F1 progeny strains of *Volvox reticuliferus* (Table 1). Parental strains are 2013-0703-VO2 (VO-2) and 2013-0703-VO3 (VO-3). F1 progeny strains are VO123-F1-6 (F1-6), VO123-F1-7 (F1-7), VO123-F1-9 (F1-9), VO123-F1-10 (F1-10). (F): Female, (M):Male strain.
(DOCX)

S5 Fig Southern blot analysis of *Volvox reticuliferus* and *V*. *africanus*. Restriction enzyme digested genomic DNA was electrophoresed on an agarose gel and stained with ethidium bromide. The corresponding Southern blot data are shown in the upper panels. A. Southern blotting using a *VrMID* fragment, located in exon1-exon3 as shown in Fig 2B. B. Southern blotting using an *EF1-like* fragment (control). Lane M contains One Step Marker 6 (Nippon Gene, Toyama, Japan) as a DNA size marker.
(DOCX)

S6 Fig DAPI staining for estimating comparative genome size in *Volvox africanus* and *V*. *reticuliferus*. A-C. Mean gray value of ten nuclei with imageJ at 0.5, 0.67, 1.0, 1.5, 2.0, 2.5 s exposure time. Bars show means and standard deviations. ctrl: *V*. *carteri* EVE strain in the same slide for control. A. *V*. *africanu*s, B. *V reticuliferus* male strain, C. *V reticuliferus* female strain. D, E. DAPI stained somatic cell of *V*. *africanus*. Scale bar = 5 μm. D. DIC image, E. DAPI stained image. Arrowhead indicates the nucleus. Yellow ring shows the region of measurement in image J. F. Fluorescence of stained somatic cell nuclei in *V*. *africanus* and *V*. *reticuliferus* relative to *V*. *carteri* EVE strain (control) at 1.5 s exposure time. Bars show means and standard deviations of 10 biological replicates.
(DOCX)

S7 Fig Alternative splicing variants of the *Volvox africanus MID* ortholog (*VaMID*). Variants 2–4 are intron retention.
(DOCX)

S1 Table Degenerate primers used in this study. (DOCX)

S2 Table Gene specific primers used in this study. (DOCX)

S3 Table List of Volvocales included in the phylogenetic analyses of MID sequences and DDBJ/EMBL/GENBANK accession numbers. (DOCX)

S4 Table Conditions for PCR cycles and primers used in semi-quantitative RT- PCR analyses (Fig 5). (DOCX)
