# Supplementary material for: Molecular evolutionary analysis of a gender-limited MID ortholog from the homothallic species Volvox africanus with male and monoecious spheroids
Source: PLoS One. 2017 Jun 30;12(6):e0180313. doi: 10.1371/journal.pone.0180313 (PMC5493378; doi:10.1371/journal.pone.0180313)
Supplement: S1 Fig — Contributions in Phycology. Allen Press, pp. 59–66). (DOCX) [file pone.0180313.s001.docx]

**
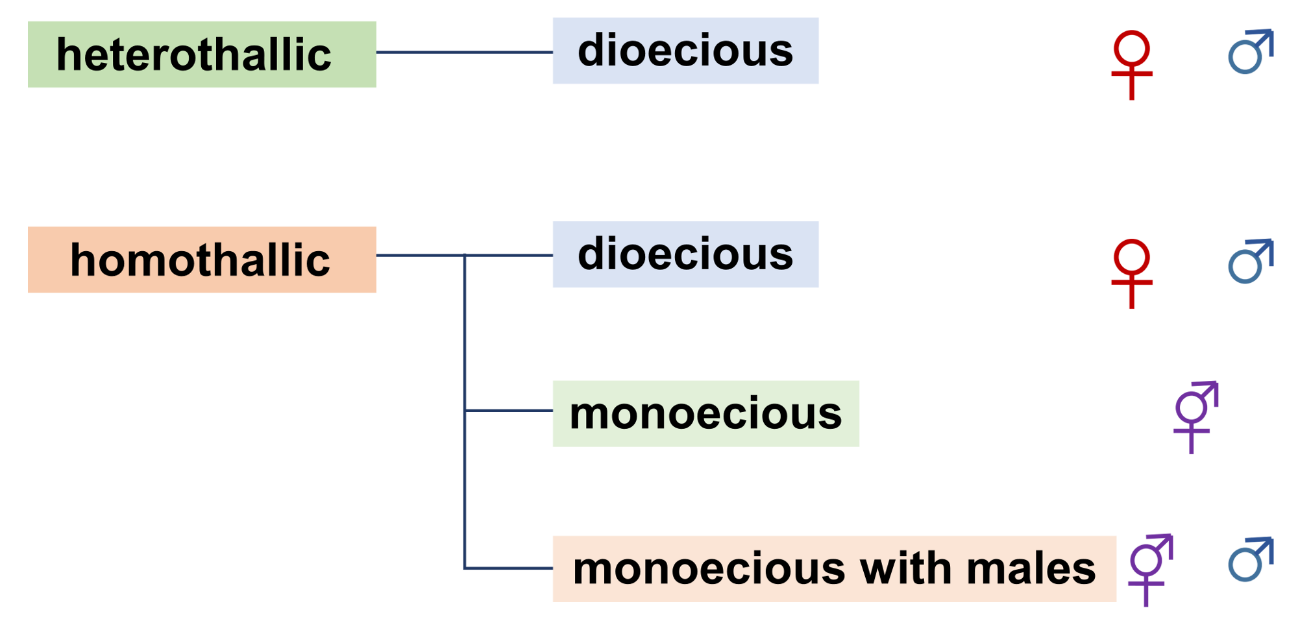
**

**S1 Fig. The four sexual types of *Volvox africanus*-like algae recognized by Starr (1971, Sexual reproduction in *Volvox africanus*. Contributions in Phycology. Allen Press, pp. 59–66).**
