# Supplementary material for: Molecular evolutionary analysis of a gender-limited MID ortholog from the homothallic species Volvox africanus with male and monoecious spheroids
Source: PLoS One. 2017 Jun 30;12(6):e0180313. doi: 10.1371/journal.pone.0180313 (PMC5493378; doi:10.1371/journal.pone.0180313)
Supplement: S2 Fig — Scale bars = 50 μm. sp: sperm packet, e: egg. A-C. V. africanus strain 2013-0703-VO4. A. Asexual spheroid. B. Monoecious spheroid. C. Male spheroid. D, E. V. reticuliferus. D. Male spheroid in male strain VO123-F1-7. E. Female spheroid in female strain VO123-F1-6. (DOCX) [file pone.0180313.s002.docx]

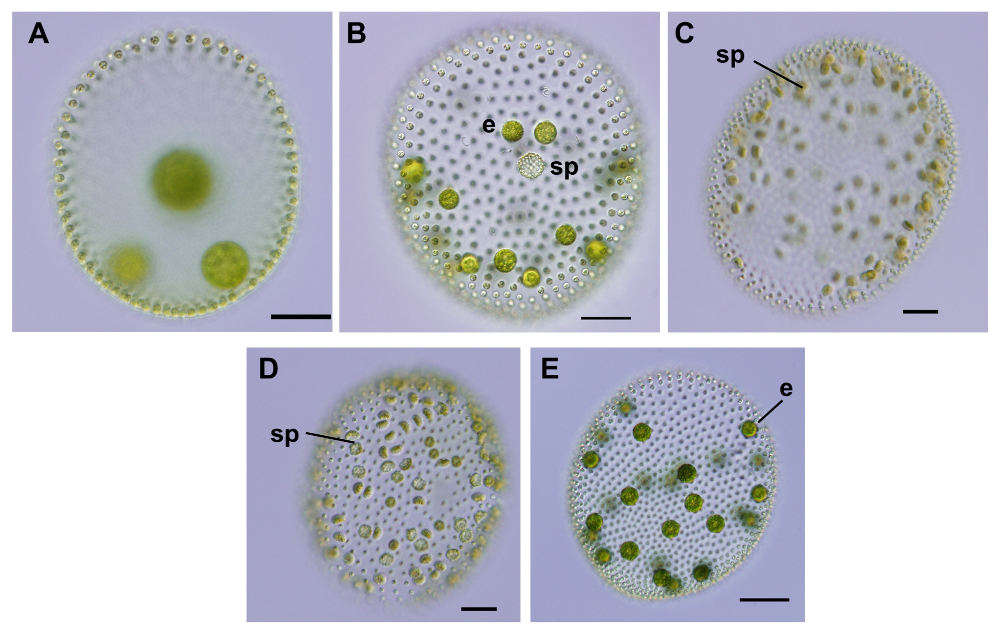


**S2 Fig. Light microscopic images of *Volvox africanus* (homothallic, monoecious with males type) and *V. reticuliferus* (heterothallic, dioecious type).**

Scale bars = 50 μm. sp: sperm packet, e: egg. A-C. *V. africanus* strain 2013-0703-VO4. A. Asexual spheroid. B. Monoecious spheroid. C. Male spheroid. D, E. *V. reticuliferus*. D. Male spheroid in male strain VO123-F1-7. E. Female spheroid in female strain VO123-F1-6.
