# Supplementary material for: Molecular evolutionary analysis of a gender-limited MID ortholog from the homothallic species Volvox africanus with male and monoecious spheroids
Source: PLoS One. 2017 Jun 30;12(6):e0180313. doi: 10.1371/journal.pone.0180313 (PMC5493378; doi:10.1371/journal.pone.0180313)
Supplement: S3 Fig — Black or gray back colors indicate over 70% of identity or similarity, respectively. (DOCX) [file pone.0180313.s003.docx]

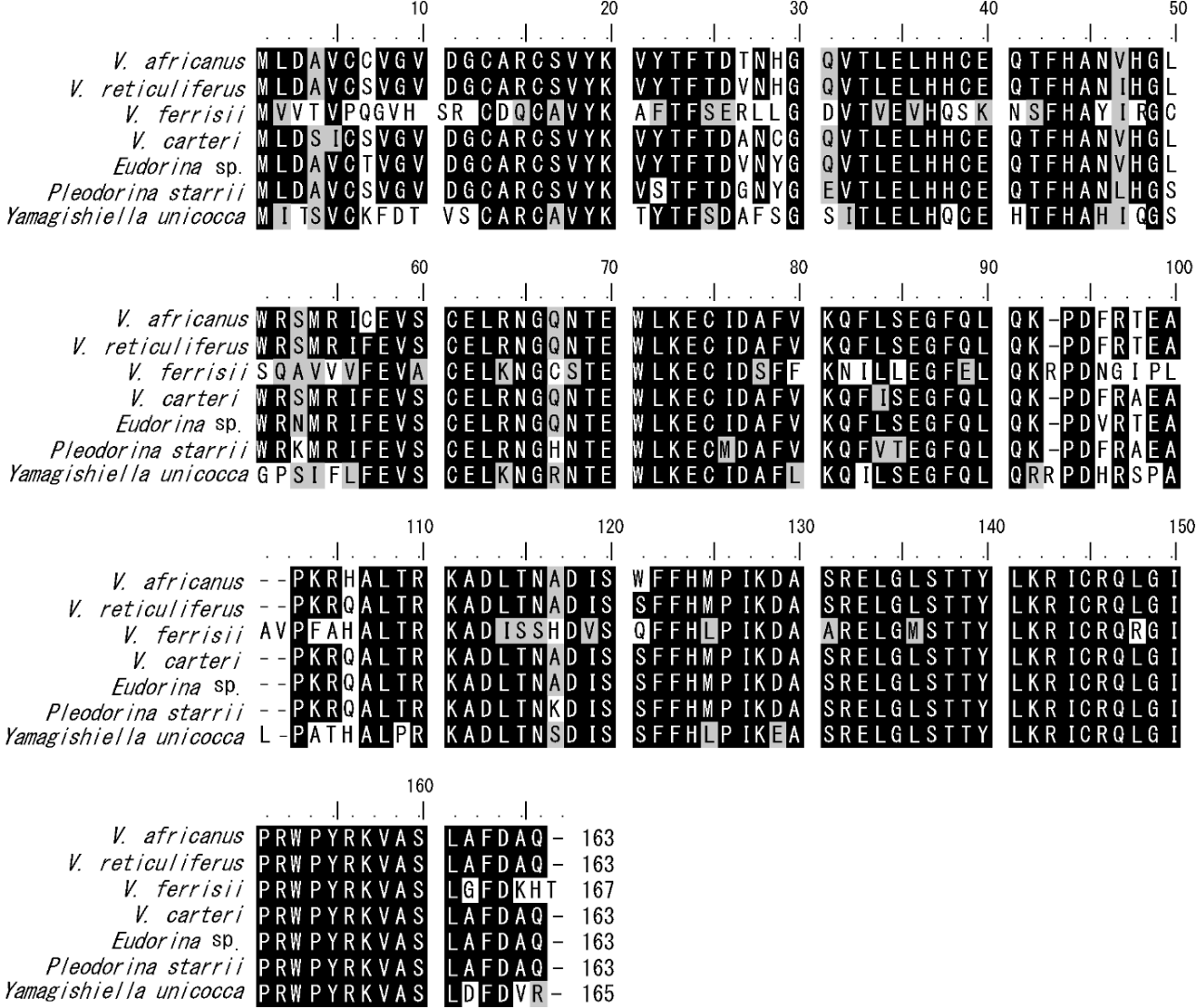


**S3 Fig. Alignment of seven MID homologs from the Volvocaceae (*Volvox africanus, V. reticuliferus, V. ferrisii, V. carteri, Eudorina* sp*., Pleodorina starrii,* and *Yamagishiella unicocca*).**

Black or gray/black back colors indicate over 70% of identity or similarity, respectively.
