# Supplementary material for: Molecular evolutionary analysis of a gender-limited MID ortholog from the homothallic species Volvox africanus with male and monoecious spheroids
Source: PLoS One. 2017 Jun 30;12(6):e0180313. doi: 10.1371/journal.pone.0180313 (PMC5493378; doi:10.1371/journal.pone.0180313)
Supplement: S4 Fig — Parental strains are 2013-0703-VO2 (VO-2) and 2013-0703-VO3 (VO-3). F1 progeny strains are VO123-F1-6 (F1-6), VO123-F1-7 (F1-7), VO123-F1-9 (F1-9), VO123-F1-10 (F1-10). (F): Female, (M):Male strain. (DOCX) [file pone.0180313.s004.docx]

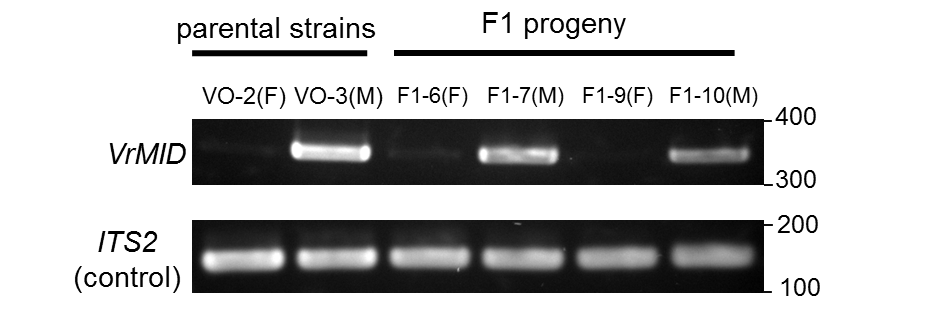
**S4 Fig. Results of genomic PCR for parental strains and four F1 progeny strains of *Volvox reticuliferus* (Table 1).** Parental strains are 2013-0703-VO2 (VO-2) and 2013-0703-VO3 (VO-3). F1 progeny strains are VO123-F1-6 (F1-6), VO123-F1-7 (F1-7), VO123-F1-9 (F1-9), VO123-F1-10 (F1-10). (F): Female, (M):Male strain.
