# Supplementary material for: Molecular evolutionary analysis of a gender-limited MID ortholog from the homothallic species Volvox africanus with male and monoecious spheroids
Source: PLoS One. 2017 Jun 30;12(6):e0180313. doi: 10.1371/journal.pone.0180313 (PMC5493378; doi:10.1371/journal.pone.0180313)
Supplement: S5 Fig — Restriction enzyme digested genomic DNA was electrophoresed on an agarose gel and stained with ethidium bromide. The corresponding Southern blot data are shown in the upper panels. A. Southern blotting using a VrMID fragment, located in exon1-exon3 as shown in Fig 2B. B. Southern blotting using an EF1-like fragment (control). Lane M contains One Step Marker 6 (Nippon Gene, Toyama, Japan) as a DNA size marker. (DOCX) [file pone.0180313.s005.docx]

**
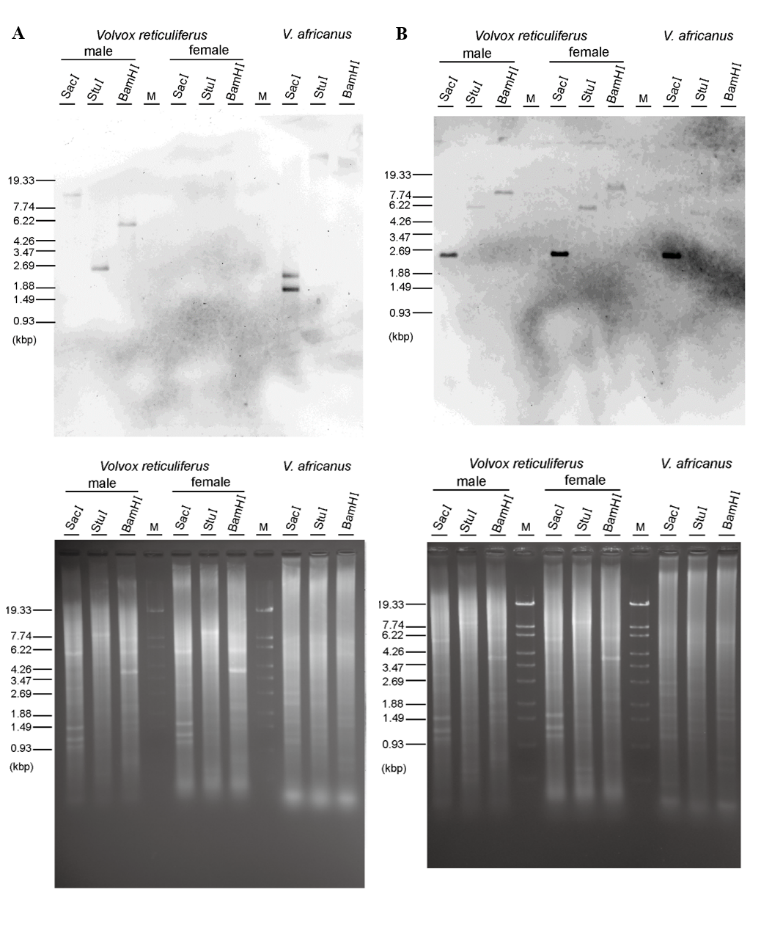
S5 Fig. Southern blot analysis of *Volvox reticuliferus* and *V. africanus*.**

Restriction enzyme digested genomic DNA was electrophoresed on an agarose gel and stained with ethidium bromide. The corresponding Southern blot data are shown in the upper panels. A. Southern blotting using a *VrMID* fragment, located in exon1-exon3 as shown in Fig. 2B. B. Southern blotting using an *EF1-like* fragment (control). Lane M contains One Step Marker 6 (Nippon Gene, Toyama, Japan) as a DNA size marker.
