# Supplementary material for: Molecular evolutionary analysis of a gender-limited MID ortholog from the homothallic species Volvox africanus with male and monoecious spheroids
Source: PLoS One. 2017 Jun 30;12(6):e0180313. doi: 10.1371/journal.pone.0180313 (PMC5493378; doi:10.1371/journal.pone.0180313)
Supplement: S6 Fig — A-C. Mean gray value of ten nuclei with imageJ at 0.5, 0.67, 1.0, 1.5, 2.0, 2.5 s exposure time. Bars show means and standard deviations. ctrl: V. carteri EVE strain in the same slide for control. A. V. africanus, B. V reticuliferus male strain, C. V reticuliferus female strain. D, E. DAPI stained somatic cell of V. africanus. Scale bar = 5 μm. D. DIC image, E. DAPI stained image. Arrowhead indicates the nucleus. Yellow ring shows the region of measurement in image J. F. Fluorescence of stained somatic cell nuclei in V. africanus and V. reticuliferus relative to V. carteri EVE strain (control) at 1.5 s exposure time. Bars show means and standard deviations of 10 biological replicates. (DOCX) [file pone.0180313.s006.docx]

**
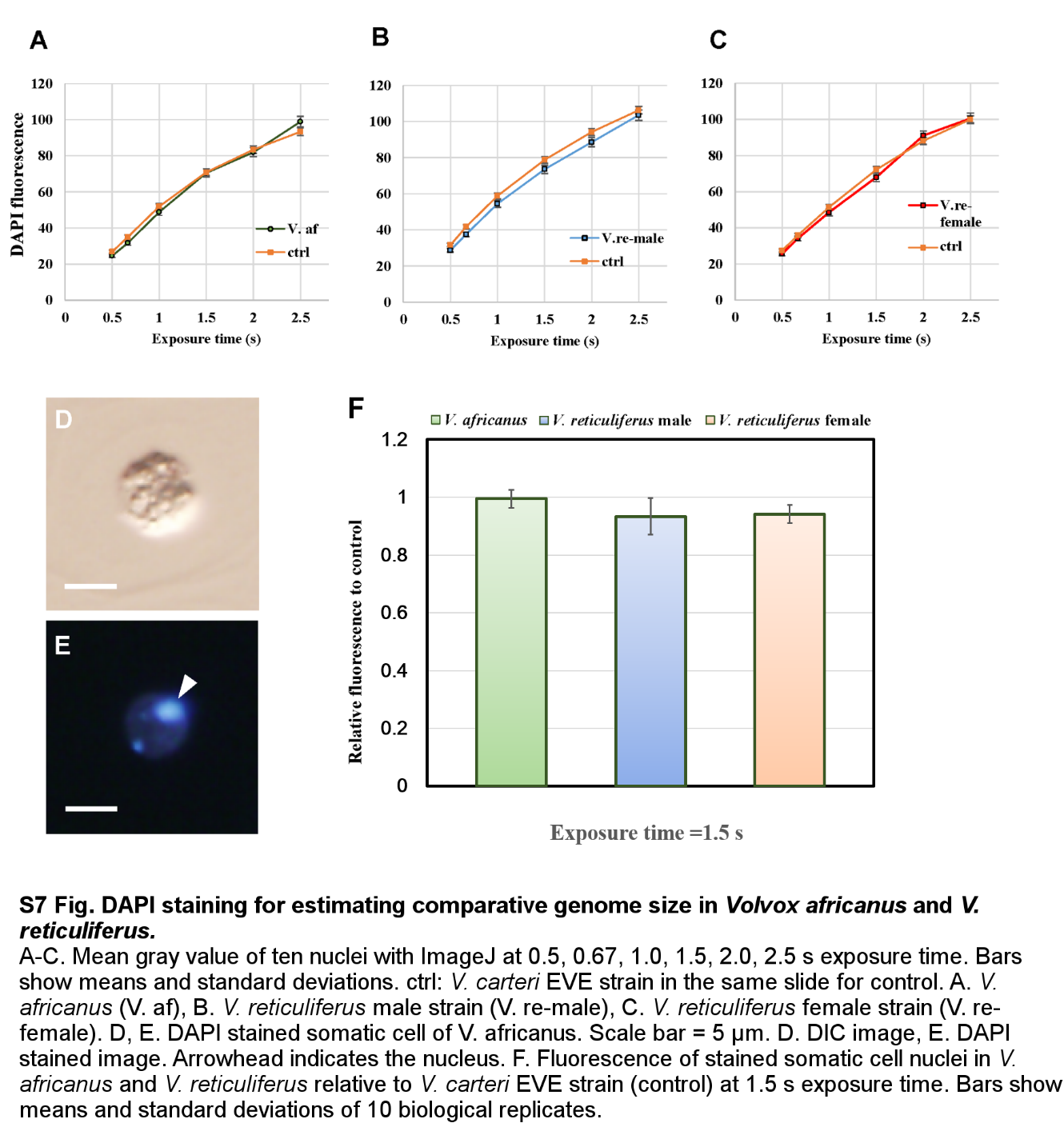
S6 Fig. DAPI staining for estimating comparative genome size in *Volvox africanus* and *V. reticuliferus*.**

A-C. Mean gray value of ten nuclei with imageJ at 0.5, 0.67, 1.0, 1.5, 2.0, 2.5 s exposure time. Bars show means and standard deviations. ctrl: *V. carteri* EVE strain in the same slide for control. A. *V. africanu*s, B. *V reticuliferus* male strain, C. *V reticuliferus* female strain. D, E. DAPI stained somatic cell of *V. africanus*. Scale bar = 5 μm. D. DIC image, E. DAPI stained image. Arrowhead indicates the nucleus. Yellow ring shows the region of measurement in image J. F. Fluorescence of stained somatic cell nuclei in *V. africanus* and *V. reticuliferus* relative to *V. carteri* EVE strain (control) at 1.5 s exposure time. Bars show means and standard deviations of 10 biological replicates.
