# Supplementary material for: Molecular evolutionary analysis of a gender-limited MID ortholog from the homothallic species Volvox africanus with male and monoecious spheroids
Source: PLoS One. 2017 Jun 30;12(6):e0180313. doi: 10.1371/journal.pone.0180313 (PMC5493378; doi:10.1371/journal.pone.0180313)
Supplement: S7 Fig — Variants 2–4 are intron retention. (DOCX) [file pone.0180313.s007.docx]

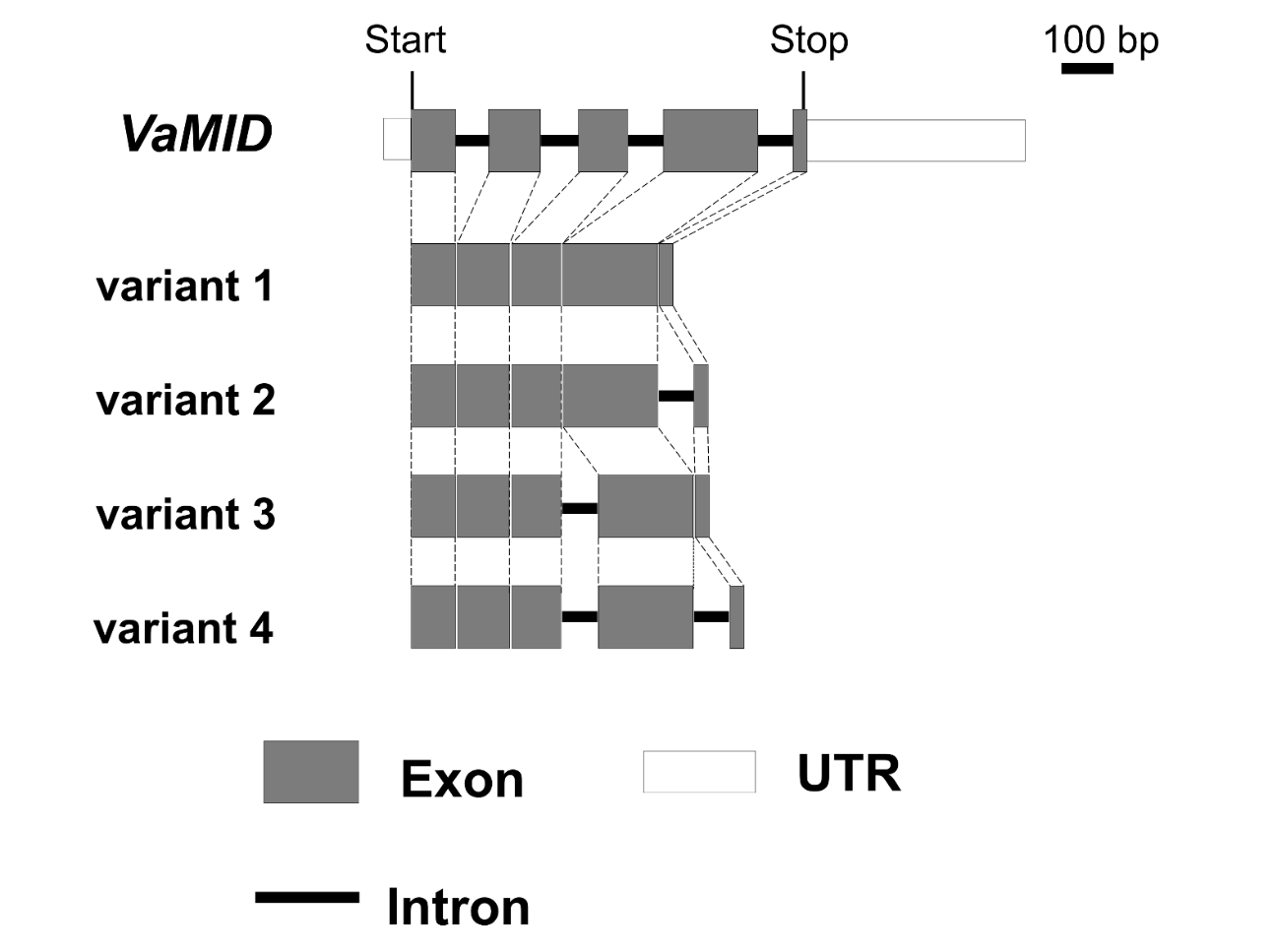
**S7 Fig. Alternative splicing variants of the *Volvox africanus MID* ortholog (*VaMID*)*.***

Variants 2-4 are intron retention.
