# Supplementary material for: Molecular evolutionary analysis of a gender-limited MID ortholog from the homothallic species Volvox africanus with male and monoecious spheroids
Source: PLoS One. 2017 Jun 30;12(6):e0180313. doi: 10.1371/journal.pone.0180313 (PMC5493378; doi:10.1371/journal.pone.0180313)
Supplement: S1 Table — (DOCX) [file pone.0180313.s008.docx]

**S1 Table. Degenerate primers used in this study.**

| **Primer name** | **Primer sequence (5' to 3')** | **forward (F) or** |
| --- | --- | --- |
|  |  | **reverse (R) primer** |
| SMID-F1 | ACIGARTGGYTIAARGAITG | F |
| SMID-R4 | GCITCYTTDATIGGIARRTG | R |
| SMID-R5 | GGDATICCIARYTGICKRCA | R |
| SMID-R6 | GCIACYTTICKRTAIGGCCA | R |
| dMT-dF3 ^a^ | RCIMRIAARGCIGAYYTIAC | F |

^a^ Hamaji et al.[1]

**Reference**

1. Hamaji T, Ferris PJ, Coleman AW, Waffenschmidt S, Takahashi F, Nishii I, et al. Identification of the minus-dominance gene ortholog in the mating-type locus of *Gonium pectorale*. Genetics 2008;178: 283–294. doi:10.1534/genetics.107.078618
