# Supplementary material for: Molecular evolutionary analysis of a gender-limited MID ortholog from the homothallic species Volvox africanus with male and monoecious spheroids
Source: PLoS One. 2017 Jun 30;12(6):e0180313. doi: 10.1371/journal.pone.0180313 (PMC5493378; doi:10.1371/journal.pone.0180313)
Supplement: S2 Table — (DOCX) [file pone.0180313.s009.docx]

**S2 Table. Gene specific primers used in this study.**

| **Primer name** | **Primer sequence (5' to 3')** | **forward (F) or reverse (R) primer** | **Gene** |
| --- | --- | --- | --- |
| VaMID_F1 | ACGCGGATATTAGCTGGTTCTTCC | F | *VaMID* |
| VaMID_F2 | TGCCAATTAAAGACGCCTCCAGAG | F |  |
| VaMID_F3 | TGGGGCTTTCAACGACATATCTGA | F |  |
| VaMID5'R1 | TGCCGGCAGATACGCTTCAGATATGT | R |  |
| VaMID5'R2 | CTCTGGAGGCGTCTTTAATTGGCATGTG | R |  |
| VaMID5'R3 | TGTCGTTGAAAGCCCCAATTCTCT | R |  |
| VaMID_AR | ACCGCTCATACTTTGCCATTAGAA | F |  |
| VaMID_ValR2 | AATACAGTCGCTCCCAAGATGAAA | R |  |
| VfMID_F1 | TTGGAAGGATTCGAGCTACAGAAG | F | *VfMID* |
| VfMID_F2 | CATACCTCTTGCAGTGCCGTTCG | F |  |
| VfMID_F3 | AAGGCCGACATCTCAAGTCACGAC | F |  |
| VfMID_F4 | GGTCGCTAGCCTGGGATTTGACAA | F |  |
| VfMID_F5 | GAAACCTTACCCTTCGGCCACTTT | F |  |
| VfMID_F6 | AGGCCTGGAAAGGTTGGTGTTCTG | F |  |
| VfMID_R1 | AAAACTGGCTGACGTCGTGACTTG | R |  |
| VfMID5'R | TGCCATTGTCCGGTCGCTTCTGTAG | R |  |
| VfMID_Rsp2 | CCGCGTTGCCGGCATATTCGCTT | R |  |
| VfMID_AF | AGCAGGTTACGTAGACTGAAGCACA | F |  |
| VfMID_AR | GTTGCCTGGGTTGTGATGCGCGTT | R |  |
| F1-7MID_3'F1 | GGACTTTGGCGTTCTATGCGGATT | F | *VrMID* |
| F1-7MID_3'F2 | GACACGCAAAGCAGACCTAACGAA | F |  |
| F1-7MID_R1 | GTCCGCGTTCGTTAGGTCTGCT | R |  |
| F1-7MID_R2 | TTAGGTCTGCTTTGCGTGTCAA | R |  |
| F1-7MID_AF | TGAAATAAGACATACGCCGGTTTCG | F |  |
| F1-7MID_AR | TGCGAAAATTCACACCGGTATGCA | R |  |
| F1-7_R3 | TCGTTAGGTCTGCTTTGCGTGTCA | R |  |
| F1-7MID_E1_F | CAGTTTGTTCTGTTGGCGTCGAC | F |  |
| F1-7_s_MID_R | TCGTTAGGTCTGCTTTGCGTGTC | R |  |
| F1-7_ITS2_F | TCCTTCTTCTTGCATGTGGATATGGC | F | *ITS2* of |
| F1-7_ITS2_R | TTAACCGAACAACTTGTGCACCCA | R | 2013-0703-VO2^a^ |
| CV_EF1A1-R2^b^ | CACGCTCGCCTGATCAACCTGCTG | F | *EF1-like* |
| GpEF1A-INT3-R^b^ | GTCCAGACCCTTGATGTTCATGCC | R |  |
| VaEF1_F2 | TTGAACTTGTTCGTCACGGTGCCT | F | *EF1-like* |
| VaEF1_R2 | TCGACAGTGAAGACCTTGCCAGTG | R |  |
| F1-7EF1_F | TCAACTTGAAGGGCGAGAAGGTCA | F |  |
| F1-7EF1_R | GTCATTTTCCTGCCCACTCACACC | R |  |
| VfEF1_F2 | AAGGAGCGCTACGATGAGATTGCC | F |  |
| VfEF1_R2 | TACCACGGCATGTTCTTGGACTCG | R |  |

^a^ Nozaki et al.[1]. ^b^ Hamaji et al.[2].

**Reference**

1. Nozaki H, Matsuzaki R, Yamamoto K, Kawachi M, Takahashi F. Delineating a new heterothallic species of *Volvox* (Volvocaceae, Chlorophyceae) using new strains of “*Volvox africanus*.” PLoS ONE. 2015;10: e0142632. doi:10.1371/journal.pone.0142632

2. Hamaji T, Ferris PJ, Coleman AW, Waffenschmidt S, Takahashi F, Nishii I, et al. Identification of the minus-dominance gene ortholog in the mating-type locus of *Gonium pectorale*. Genetics 2008;178: 283–294. doi:10.1534/genetics.107.078618
