# Supplementary material for: Molecular evolutionary analysis of a gender-limited MID ortholog from the homothallic species Volvox africanus with male and monoecious spheroids
Source: PLoS One. 2017 Jun 30;12(6):e0180313. doi: 10.1371/journal.pone.0180313 (PMC5493378; doi:10.1371/journal.pone.0180313)
Supplement: S3 Table — (DOCX) [file pone.0180313.s010.docx]

**S3 Table. List of Volvocales included in the phylogenetic analyses of MID sequences and DDBJ/EMBL/GENBANK accession numbers.**

| **Species** | **Strain designation** | **Accession number** | **Reference** |
| --- | --- | --- | --- |
| *V. africanus* | 2013-0703-VO4 | LC274875, LC274876 | The present study |
|  | (=NIES-3780) |  |  |
| *V. reticuliferus* | VO123-F1-7 | LC274879, LC274880 | The present study |
|  | (=NIES-3786) |  |  |
| *V. ferrisii* | 2011-929-Vx2-F2-9 | LC274877, LC274878 | The present study |
|  | (=NIES-3986) |  |  |
| *V. carteri* | UTEX 1886 | GU784916.1 | Ferris et al. [1] |
| *Pleodorina starrii* | NIES-1363 | BAF42661 | Nozaki et al. [2] |
| *Yamagishiella unicocca* | NIES-1859 | LC274882 | The present study |
| *Eudorina* sp. 2006-703-Eu-15 | NIES-2735 | LC274881 | The present study |
| *G. maiaprilis* | NIES-2457 | AB623044 | Setohigashi et al. [3] |
| *G. multicoccum* (homothallic) | NIES-1708 | AB774226 | Hamaji et al. [4] |
| *G. multicoccum* (heterothallic) | NIES-1038 | AB774225 | Hamaji et al. [4] |
| *G. octonarium* | NIES-852 | AB774227 | Hamaji et al. [4] |
| *G. pectorale* | NIES-1710 | AB353340 | Hamaji et al. [2] |
| *G. quadratum* | NIES-652 | AB774228 | Hamaji et al. [4] |
| *G. viridistellatum* | NIES-654 | AB774224 | Hamaji et al. [4] |
| *Chlamydomonas reinhardtii* | CC-621 | AAC49753 | Ferris and Goodenough [5] |
| *C. globosa* | CC-1870 | AAB60944.1 | Ferris et al. [6] |

**References**

1. Ferris P, Olson BJSC, De Hoff PL, Douglass S, Casero D, Prochnik S, et al. Evolution of an expanded sex-determining locus in *Volvox*. Science 2010;328: 351–354. doi:10.1126/science.1186222

2. Hamaji T, Ferris PJ, Coleman AW, Waffenschmidt S, Takahashi F, Nishii I, et al. Identification of the minus-dominance gene ortholog in the mating-type locus of *Gonium pectorale*. Genetics 2008;178: 283–294. doi:10.1534/genetics.107.078618

3. Setohigashi Y, Hamaji T, Hayama M, Matsuzaki R, Nozaki H. Uniparental inheritance of chloroplast DNA is strict in the isogamous volvocalean *Gonium*. PLoS ONE 2011;6: e19545. doi:10.1371/journal.pone.0019545

4. Hamaji T, Ferris PJ, Nishii I, Nishimura Y, Nozaki H. Distribution of the sex-determining gene *MID* and molecular correspondence of mating types within the isogamous genus *Gonium* (Volvocales, Chlorophyta). PLoS ONE 2013;8: e64385. doi:10.1371/journal.pone.0064385

5. Ferris PJ, Goodenough UW. Mating type in *Chlamydomonas* is specified by *mid*, the minus-dominance gene. Genetics 1997;146: 859–869.

6. Ferris PJ, Pavlovic C, Fabry S, Goodenough UW. Rapid evolution of sex-related genes in *Chlamydomonas*. Proc. Natl. Acad. Sci. USA. 1997;94: 8634–8639.
