# Supplementary material for: Molecular evolutionary analysis of a gender-limited MID ortholog from the homothallic species Volvox africanus with male and monoecious spheroids
Source: PLoS One. 2017 Jun 30;12(6):e0180313. doi: 10.1371/journal.pone.0180313 (PMC5493378; doi:10.1371/journal.pone.0180313)
Supplement: S4 Table — (DOCX) [file pone.0180313.s011.docx]

**S4 Table. Conditions for PCR cycles and primers used in semi-quantitative RT- PCR analyses (Fig. 5).**

| **Species** | **gene** | **PCR cycles** | **forward (F) or** | **Primer name** | **Sequence (5' to 3')** |
| --- | --- | --- | --- | --- | --- |
|  |  |  | **reverse (R) primer** | **(S3 Table)** |  |
| ***V. africanus*** | *MID* | 2 min at 94℃, followed by 32, 34 or 36 cycles of 10 sec at 98℃, 30 sec at 65℃ and 30 sec at 68℃. | F | VaMID_AR | ACCGCTCATACTTTGCCATTAGAA |
|  |  |  | R | VaMID_ValR2 | AATACAGTCGCTCCCAAGATGAAA |
|  | *EF1-like* | 2 min at 94℃, followed by 28, 30 or 32 cycles of 10 sec at 98℃, 30 sec at 63℃ and 30 sec at 68℃. | F | VaEF1_F2 | TTGAACTTGTTCGTCACGGTGCCT |
|  |  |  | R | VaEF1_R2 | TCGACAGTGAAGACCTTGCCAGTG |
| ***V. reticuliferus* male strain** | *MID* | 2 min at 94℃, followed by 32, 34 or 36 cycles of 10 sec at 98℃ and 30 sec at 68℃. | F | F1-7MID_3'F1 | GGACTTTGGCGTTCTATGCGGATT |
|  |  |  | R | F1-7_R3 | TCGTTAGGTCTGCTTTGCGTGTCA |
|  | *EF1-like* | 2 min at 94℃, followed by 28, 30 or 32 cycles of 10 sec at 98℃ and 30 sec at 68℃. | F | F1-7EF1_F | TCAACTTGAAGGGCGAGAAGGTCA |
|  |  |  | R | F1-7EF1_R | GTCATTTTCCTGCCCACTCACACC |
| ***V. ferrisii*** | *MID* | 2 min at 94℃, followed by 32, 34 or 36 cycles of 10 sec at 98℃ and 30 sec at 68℃. | F | VfMID_F3 | AAGGCCGACATCTCAAGTCACGAC |
|  |  |  | R | VfMID_Rsp2 | CCGCGTTGCCGGCATATTCGCTT |
|  | *EF1-like* | 2 min at 94℃, followed by 28, 30 or 32 cycles of 10 sec at 98℃ and 30 sec at 68℃. | F | VfEF1_F2 | AAGGAGCGCTACGATGAGATTGCC |
|  |  |  | R | VfEF1_R2 | TACCACGGCATGTTCTTGGACTCG |
